# Baseline amplicon sequencing data for the ITS2 region in the green honey of Banggi Island, Sabah

**DOI:** 10.1016/j.dib.2024.110044

**Published:** 2024-01-09

**Authors:** Saeed ullah, Nurul Huda, Roswanira Ab. Wahab, Azzmer Azzar Abdul Hamid, Mohd Hamzah Mohd Nasir, Mohd Azrul Naim Mohamad, Hajar Fauzan Ahmad, Habeebat Adekilekun Oyewusi, Fahrul Huyop

**Affiliations:** a Department of Biosciences, Faculty of Science, Universiti Teknologi Malaysia, 81310 Johor Bahru, Malaysia; b Faculty of Sustainable Agriculture, Universiti Malaysia Sabah, 90509 Sandakan, Sabah, Malaysia; c Department of Chemistry, Faculty of Science, Universiti Teknologi Malaysia, 81310 Johor Bahru, Malaysia; d Research Unit for Bioinformatics and Computational Biology (RUBIC), Kulliyyah of Science, International Islamic University Malaysia, Bandar Indera Mahkota, 25200 Kuantan, Pahang, Malaysia; e Faculty of Industrial Sciences and Technology, Universiti Malaysia Pahang Al Sultan Abdullah, 26300 Gambang, Pahang, Malaysia; f Department of Biochemistry, School of Science and Computer Studies, Federal Polytechnic Ado Ekiti, Ado Ekiti PMB 5351, Ekiti State, Nigeria

**Keywords:** Green honey, ASVs, eDNA, Illumina sequencing, Internal Transcribe Spacer 2, Banggi Island

## Abstract

Green honey, was discovered on Banggi Island, Sabah, showing high in essential amino acids and chlorophyll derivatives. Despite its lucrative market potential owing to its distinctive color, uncertainties persist regarding its nature. This study leverages amplicon sequencing by targeting micro- and macro-organisms present in honey environmental DNA (eDNA) using Internal Transcribed Spacer 2 (ITS2) region, enabling the identification of floral and microorganism sources that represent the honey's composition. The investigation into green honey from Banggi Island concerns the prevalence of honey adulteration and authenticity for economic gain. Adulteration methods, such as the addition of sugar syrups, compromise honey purity. Using a sequencing approach would help in determining the geographic origin and verifying the authenticity of the honey. The study aims to identify plant species or microorganisms in honey's eDNA. To authenticate honey, we utilized ITS2 with Illumina sequencing, exploring the diversity of green honey samples. Raw sequence reads obtained for the green honey sample revealed 1,438,627 raw reads, with a GC average of 49.22 %. A total of 44 amplicon sequence variances (ASVs) were identified, including three genera: *Zygosaccharomyces* with two species, *Fraxinus* with three species, and the genus *Ficaria* with only one species. Their respective relative abundances were 98.55%, 0.94%, and 0.51%. *Zygosaccharomyces rouxii* and *Zygosaccharomyces mellis* were identified as the pre-dominant yeast species in honey, while the *Fraxinus* and *Ficaria* genus represent common plant species in Sabah, particularly in Banggi Island. The dominance of *Zygosaccharomyces* species aligns with their known prevalence in honey, affirming the reliability of our findings. The presence of *Fraxinus* and *Ficaria* in the honey sample correlates with its abundance in the local environment. This amplicon sequencing approach not only contributes to our understanding of green honey composition but also serves as a valuable resource for authenticating honey origin in Malaysia, particularly for green honey from Banggi Island, Sabah. Our study pioneers the application of ITS2 amplicon sequencing for green honey amplicon sequencing, providing valuable insights into its composition and origin. This methodology, with a focus on eDNA, contributes to the authentication and quality determination of honey in Malaysia, addressing the pressing concerns of adulteration and variability in production practices.

Specifications Table

**Table:** 

Subject	Molecular biology
Specific subject area	Microbiome, amplicon sequencing
Data format	Raw, filter, assembled and analyzed
Type of data	Table, Figure, Fasta file
Data collection	The green honey sample extraction, amplification, sequencing, and subsequent analysis of the eDNA for fungi and plant species were performed successively.
Data source location	Raw green honey sample collected from Banggi Island Sabah (Lat. 7.211973, Long. 117.121943). The collected sample were analysed in: Universiti Teknologi Malaysia, Johor Bahru, Malaysia
Data accessibility	Repository name: National Center for Biotechnology Information (NCBI) Sequence Read Archive (SRA) data: Accession number: SRR26876357GH ITS2 and PRJNA1041960 Direct Link: https://ncbi.nlm.nih.gov/bioproject/PRJNA1041960 Identification of Fungi/yeast and plant species in green honey Revolved Honey diversity and origin (NCBI).
Related research article	Huda, N., ullah, S., Wahab, R. A., Lani, M. N., Daud, N. H. A., Shariff, A. H. N. Ismail, A. Abdul Hamid, M. A. Naim Mohamad, Huyop, F. The first ITS2 sequence data set of eDNA from honey of Malaysian giant honeybees (*Apis dorsata*) and stingless bees (*Heterotrigona itama*) reveals plant species diversity. BMC Research Notes, 16(1), (2023) 211. https://doi.org/10.1186/s13104-023-06495-9 [1]

## 1 Value of the Data

• The data article represents the baseline data set of ITS2 for green honey samples.
• The dominant fungi/yeast and plant genera present in green honey sample.
• The data article helps understand the true origin and authentication of green honey samples.

## 2 Background

The discovery of green honey has sparked inquiries into the authenticity and prevalence of honey adulteration on Banggi Island. It raises questions about the source, as well as the botanical and environmental factors contributing to this unusual coloration. To address these uncertainties and shed light on the intriguing phenomenon of green honey, this study aims to investigate details of its composition. The primary objective of this research is to elucidate the floral components that contribute to the unique color and flavor profile of green honey. Furthermore, the study will investigate the presence of microorganisms within the honey matrix, providing insights into the ecological aspects of honey production on Banggi Island.

## 3 Data Description

The data set contains a fungi /yeast microbiome and plant species profiles detected in green honey samples from Banggi Island. The FASTA files served as the primary source of metadata for the bioinformatics analysis conducted in this work. The raw fungi/yeast and Plant FASTA files of the green honey sample are made accessible via the National Centre for Biotechnology Information (NCBI) data repository system. The ITS2 together with alumina sequencing identified 1,438,627 raw reads and an average GC percentage of 49.22 and 44 ASVs identified were created for green honey samples (Table 1).

**Table 1:** General features of eDNA green honey predicted by NCBI genome annotation pipeline.

Attribute	eDNA green honey
Total bases generated (bp)	362,574,750
Processed reads	1,216,480
Filter reads	1,211,585
Number of OTUs identified	44
N_50_ (bp) of OTUs	15,534
G + C content (%) of OTUs	49.22
Sequence Read Archive	PRJNA1041960
BioSample accession	SAMN38304765
BioProject accession	PRJNA1036045

Table 2 An overview of the alpha diversity indices for the limited amount of eDNA green honey namely ace, Chao 1, Faith-pd, obsfeet, Shannon, and Simpson. This is to demonstrate the diversity of yeast/fungi and plant species found in the green honey.

**Table 2:** Alpha diversity of fungi/yeast and plant community.

Sample	Ace	Chao1	Faith-pd	Obsfeet	Shannon	Simpson
GH	43	43	0.32	43	1.59	0.43

**Fig. 1:**
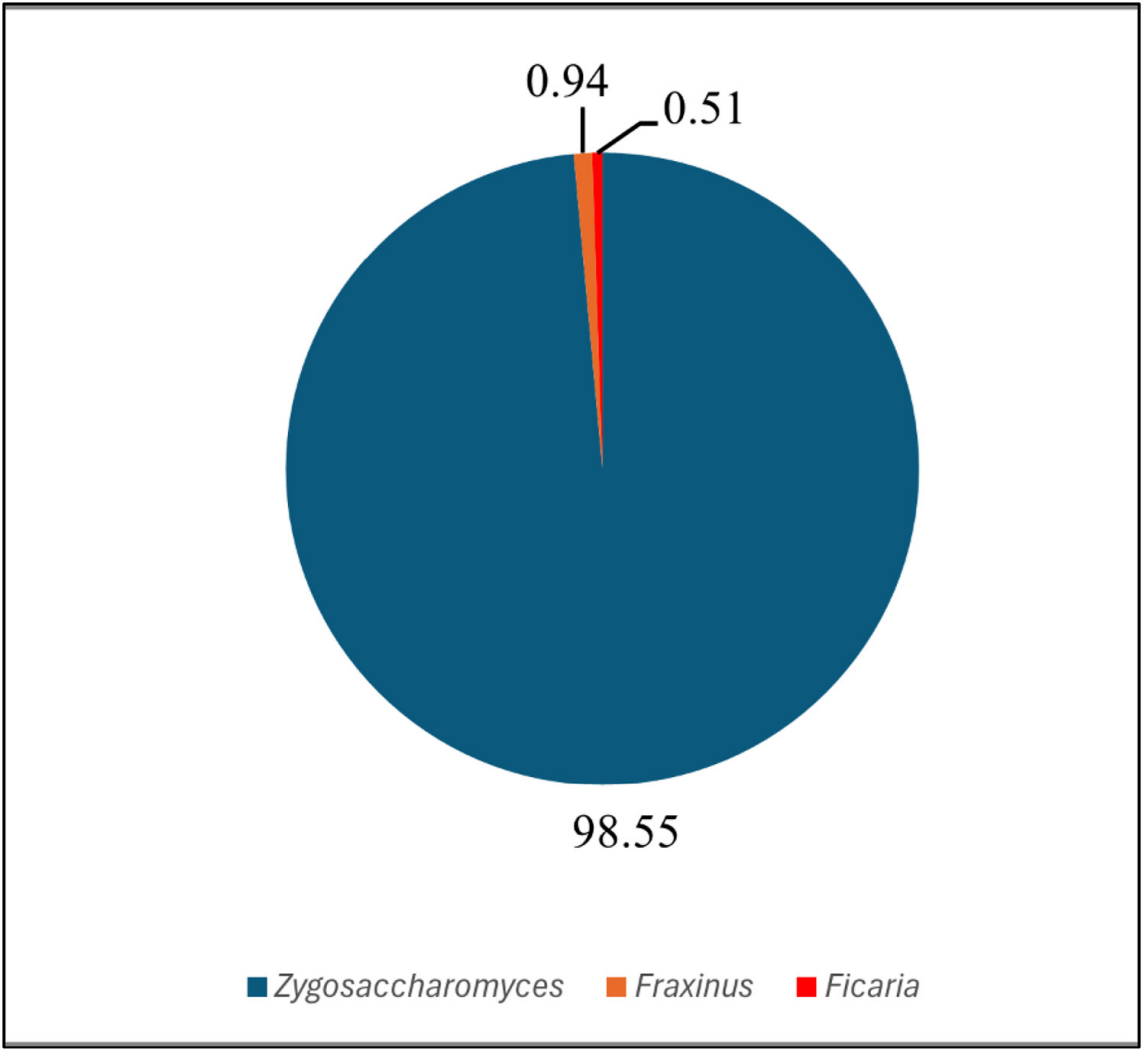
Relative abundance of genera identified in a green honey eDNA.

Fig 1 illustrates the relative abundance of identified genera in a green honey sample, presenting a pie chart that visually represents the distribution of these genera. The chart provides the diversity of the eDNA green honey ecosystem. Table 3 presents counts for all classifications (Phylum, Class, Order, Family, and Genus), accompanied by the relative abundance of each fungi/yeast taxon identified in green honey. In contrast, Table 4, displays counts for all classifications and the relative abundance of identified plant species. The current data highlights novel observations and the authenticity of ecological diversity in Banggi Island's eDNA green honey. It includes the relative abundance of each taxon in both plant and fungus/yeasts communities.

**Table 3:** Yeast classifications (Phylum, Class, Order, Family, and Genus) identified from eDNA green honey.

OTU	Phylum	Class	Order	Family	Genus / species	RA (%)
ASV00001	Ascomycota	Saccharomyces	Saccharomycetales	Saccharomycetaceae	*Zygosaccharomyces rouxii*	74.403
ASV00002	Ascomycota	Saccharomyces	Saccharomycetales	Saccharomycetaceae	*Zygosaccharomyces mellis*	8.837
ASV00003	Ascomycota	Saccharomyces	Saccharomycetales	Saccharomycetaceae	*Zygosaccharomyces mellis*	8.585
ASV00004	Ascomycota	Saccharomyces	Saccharomycetales	Saccharomycetaceae	*Zygosaccharomyces mellis*	1.632
ASV00005	Ascomycota	Saccharomyces	Saccharomycetales	Saccharomycetaceae	*Zygosaccharomyces rouxii*	0.741
ASV00006	Ascomycota	Saccharomyces	Saccharomycetales	Saccharomycetaceae	*Zygosaccharomyces rouxii*	0.654
ASV00007	Ascomycota	Saccharomyces	Saccharomycetales	Saccharomycetaceae	*Zygosaccharomyces rouxii*	0.981
ASV00008	Ascomycota	Saccharomyces	Saccharomycetales	Saccharomycetaceae	*Zygosaccharomyces rouxii*	0.598
ASV00009	Ascomycota	Saccharomyces	Saccharomycetales	Saccharomycetaceae	*Zygosaccharomyces mellis*	0.582
ASV00010	Ascomycota	Saccharomyces	Saccharomycetales	Saccharomycetaceae	*Zygosaccharomyces mellis*	0.702
ASV00011	Ascomycota	Saccharomyces	Saccharomycetales	Saccharomycetaceae	*Zygosaccharomyces rouxii*	0.487
ASV00013	Ascomycota	Saccharomyces	Saccharomycetales	Saccharomycetaceae	*Zygosaccharomyces mellis*	0.396
ASV00014	Ascomycota	Saccharomyces	Saccharomycetales	Saccharomycetaceae	*Zygosaccharomyces rouxii*	0.343

**Table 4:** Numbers of plant species identified from eDNA green honey.

OTU	Phylum	Class	Order	Family	Genus/species	RA (%)
ASV00001	Magnoliophyta	Magnoliopsida	Ranunculales	Ranunculaceae	*Ficaria verna*	0.51
ASV00002	Anthophyta	Dicotyledoneae	Lamiales	Oleaceae	*Fraxinus pennsylvanica*	0.23
ASV00003	Anthophyta	Dicotyledoneae	Lamiales	Oleaceae	*Fraxinus albicans*	0.30
ASV00004	Anthophyta	Dicotyledoneae	Lamiales	Oleaceae	*Fraxinus paxiana*	0.41

## 4 Experimental Design, Materials and Methods

### 4.1 Sample collection

For the analysis, raw green honey was received from NS Field Sdn. Bhd. The honey sample was harvested between the months of June and July 2023 on Banggi Island (7.211973, 117.121943) in Sabah.

### 4.2 Sample preparation

Honey is very rich of sugar content, therefore have been improvements in the procedure for DNA production. The extraction of DNA was performed with little modification as previously described [2], [3], [4]. A 50 g sample of honey was divided into four 50 mL Falcon tubes, each containing 12.5 g of honey. Subsequently, 2 mL of ultrapure water was added to each tube. The solutions were then incubated at a temperature of 10 °C for a duration of 30 min while being stirred. Following centrifugation at a force of 30,000 *g* for 20 min, the liquid portions located above the sediment were removed and discarded. The pellet was resuspended and mixed in a solution consisting of 1 mL of ultrapure water and 1 mL of phosphate-buffered saline (PBS). The resulting mixture was then transferred to a 2 mL tube and subjected to centrifugation at a force of 30,000 *g* for a duration of 20 min. The liquid portion was discarded while the solid residue was preserved at a temperature of −20 °C for DNA extraction.

### 4.3 DNA extraction

Solid residue or biomass of green honey were gathered into two centrifuge tubes as a replicate and extraction of DNA was performed using standard protocols of QIAamp® Powerful® Pro Kit (QIAGEN) with modification [5].

### 4.4 Qualitative assessment of DNA via gel electrophoresis

The extracted DNA was evaluated by observing the DNA bands using gel electrophoresis to determine its quality. A 1 × TAE (Tris-acetate-EDTA) buffer solution was prepared by stirring 25 mL of 40 × TAE buffer with 975 mL of deionized water. The resulting solution was then stored in a Scotch bottle. To prepare a 1% (w/v) agarose gel, 0.3 g of agarose powder was combined with 30 ml of 1 × TAE buffer and heated until the agarose was completely dissolved. After cooling under running tap water, the molten agarose solution was poured into an electrophoresis cast and solidified at room temperature for 20 min. subsequently, a 1:10,000 Diamond™ Nucleic Acid Dye dilution was created by mixing 0.1 µL dye with 1000 µL TAE buffer in a microcentrifuge tube. The diluted dye was stored at −20 °C for long-term storage.

To run the agarose gel, add 1 × TAE buffer to the tank until it covers the gel's surface. In the first well of the agarose gel, DNA marker was added in a 1:1 ratio with Diamond™ Nucleic Acid Dye. The lane was loaded with a combination of 1 µL of Diamond™ Nucleic Acid Dye and 5 µL of the DNA sample. In the negative control, 5 µL of the DNA sample was replaced with distilled water. The electrophoresis system was operated at a voltage gradient of 5 Vs per centimetre for a duration of 30 min. Subsequently, the gel was scrutinised using ultraviolet (UV) light in the gel documentation system (Amersham Imager 680) to analyse the DNA bands that were obtained [6].

### 4.5 Library preparation and sequencing

The plant ITS2 region was amplified using the primers ITS-S2F ATGCGATACTTGGTGTGAAT and ITS-p4 CCGCTTAKTGATATGCTTAAA [7]. An additional 5 bases of inline barcode were incorporated at the 5′ end of the primers to enable inline barcoding [8]. Different samples were amplified using different combinations of the forward and reverse inline primers. PCR was performed using Solar Bio PCR master mix (SolarBio, China) with the PCR profile of 95 °C for 3 min followed by 40 cycles of 95 °C for 15 s, 47 °C for 20, and 72 °C for 15 s.

The barcoded amplicons were subsequently visualized on gel and purified using 0.8 X of SPRI bead. The purified amplicons were used as the template for 8 cycles of index PCR to incorporate the complete Illumina adapter and Illumina-compatible dual-index barcodes. The constructed libraries were subsequently size selected using 0.8 X vol of SPRI bead and pooled into a single tube. Quantification of the pooled libraries used Denovix high sensitivity assay. Sequencing of the pooled libraries was performed on a NovaSEQ6000 (Illumina, San Diego) using the 2 × 150 bp paired-end sequencing configuration.

### 4.6 Bioinformatics and statistical analysis

Demultiplexing and primer trimming of the raw paired-end reads used cut adapt v1.18 [9]. The trimmed reads were subsequently merged using fastp v0.21 [10]. The processed reads were imported into QIIME2 v.2022.8 [11] for further analysis. Amplicon Sequence Variants (ASVs) were obtained using the dada2 v1.22 R package [12]. Taxonomic assignment of the ASVs was carried out using q2-feature-classifier [13] which has been trained on the latest UNITE database (unite_ver9_dynamic) [14]. Only ASVs with taxonomic assignment at least to the phylum level were selected for subsequent analysis.

The ASV table and taxonomic classification table were exported using QIIME2 tools into tab-separated values (TSV format) and manually formatted to generate Microbiome Analyst-compatible input [15]. This prepared data was utilized for various analyses, including SparCC co-occurrence network construction [16] and statistical analysis employing the linear discriminant analysis (LDA) effect size (LEfSe) method [17]. Alpha- and beta-diversity was calculated using specialized QIIME2 plug-ins. To gain insights into the relative abundances among taxonomic hierarchies, a filtered relative abundance table was also used as the input to generate Krona plots [18].

## Limitations

• A limited sample size may not accurately reflect the larger population and therefore restrict the applicability of the findings.
• Extraction of DNA from honey possess limitation like low biomass yield, DNA degradation and the presence of foreign DNA.
• The study specifically examines honey samples from the Banggi Island region, which may restrict the applicability of the results to other places or countries.
• The age, of honey can influence the variety and quantity of plant/fungal species found in the sample.

## Ethics Statement

The current research work does not involve studies with animals and humans.

## CRediT authorship contribution statement

**Saeed ullah:** Formal analysis, Investigation, Writing – original draft, Conceptualization, Methodology, Data curation. **Nurul Huda:** Methodology, Data curation, Supervision. **Roswanira Ab. Wahab:** Supervision, Validation, Conceptualization, Methodology. **Azzmer Azzar Abdul Hamid:** Supervision, Validation, Methodology, Data curation. **Mohd Hamzah Mohd Nasir:** Supervision, Validation, Conceptualization, Methodology. **Mohd Azrul Naim Mohamad:** Conceptualization, Methodology, Data curation. **Hajar Fauzan Ahmad:** Conceptualization, Methodology, Data curation. **Habeebat Adekilekun Oyewusi:** Supervision, Validation, Conceptualization. **Fahrul Huyop:** Supervision, Conceptualization, Formal analysis, Funding acquisition, Validation, Writing – review & editing.

## Data Availability

Identification of Fungi/yeast and plant species in green honey Revolved Honey diversity and origin (NCBI). (Original data) (SRA (NCBI))
